# Clinical and Genetic Characterization of Adolescent-Onset Epilepsy: A Single-Center Experience in Republic of Korea

**DOI:** 10.3390/biomedicines12122663

**Published:** 2024-11-22

**Authors:** Ji Yoon Han, Tae Yun Kim, Joonhong Park

**Affiliations:** 1 Department of Pediatrics, College of Medicine, The Catholic University of Korea, Seoul 06591, Republic of Korea; han024@catholic.ac.kr; 2 Department of Thoracic and Cardiovascular Surgery, College of Medicine, Jeonbuk National University, Jeonju 54907, Republic of Korea; cseokim@jbnu.ac.kr; 3 Department of Laboratory Medicine, College of Medicine, Jeonbuk National University, Jeonju 54907, Republic of Korea; 4 Research Institute of Clinical Medicine of Jeonbuk National University-Biomedical Research Institute of Jeonbuk National University Hospital, Jeonju 54907, Republic of Korea

**Keywords:** adolescent-onset epilepsy, neurodevelopmental delay, chromosomal microarray, clinical exome sequencing, genetic diagnosis

## Abstract

Objectives: This study investigated the characteristics of adolescent-onset epilepsy (AOE) and conducted genetic tests on a cohort of 76 Korean patients to identify variants and expand the spectrum of mutations associated with AOE. Methods: Clinical exome sequencing after routine karyotyping and chromosomal microarray was performed to identify causative variants and expand the spectrum of mutations associated with AOE. Results: In cases of AOE without neurodevelopmental delay (NDD), this study identified four likely pathogenic variants (LPVs) or variants of uncertain significance (VUS) and two copy number variations (CNVs). To explore the unique features of AOE; clinical manifestations were compared between patients with and without NDD. The analysis revealed statistically significant differences in the prevalence of males and the yield of genetic testing results. AOE without NDD had a lower prevalence in males (49%) compared to AOE with NDD (60%) (*p* = 0.007). Genetic alterations: AOE with NDD exhibited a higher frequency of genetic alterations (35%) compared to AOE without NDD (12%) (*p* = 0.011). Thorough evaluation of AOE can be particularly challenging in adolescent patients. Some individuals may display genetic variations due to a phenomenon known as locus heterogeneity, where different genetic causes lead to similar clinical presentations. Conclusions: Implementing a robust genetic workflow is crucial for accurately diagnosing AOE, even in cases with complex genetic underpinnings. This study underscores the importance of genetic testing as an essential diagnostic tool for AOE. Identifying genetic variants and understanding their clinical correlations can aid in improving diagnostic accuracy and optimizing treatment approaches for adolescent patients with epilepsy.

## 1. Introduction

Epilepsy is a complex neurological disorder characterized by recurrent unprovoked seizures, affecting approximately 1.5–2% of the general population worldwide [1]. While the incidence of epilepsy is highest in infancy and childhood, adolescence represents a critical period where the condition poses unique challenges [2]. Adolescent-onset epilepsy (AOE) encompasses not only epilepsy syndromes that emerge during adolescence but also those that originate earlier in life and persist into the teenage years [3]. Adolescence is a pivotal developmental stage marked by significant physical, psychological, and social changes as individuals transition toward adulthood [4,5]. During this period, adolescents strive for independence, navigate evolving social relationships, and often engage in risk-taking behaviors [5]. For those with epilepsy, these typical developmental challenges are compounded by the condition’s impact on their daily lives. The psychosocial aspects of AOE are crucial for a comprehensive understanding of the condition as they significantly influence disease management, treatment adherence, and overall quality of life [6]. Adolescents with epilepsy may face social stigma, discrimination, and isolation, which can lead to lowered self-esteem, anxiety, and depression [7,8]. The fear of having a seizure in public can restrict participation in social activities, sports, and academic endeavors, hindering normal social development [9]. Moreover, epilepsy can affect cognitive functions, academic performance, and vocational planning, further impacting an adolescent’s future opportunities [10]. The transition from pediatric to adult healthcare services also presents challenges, as adolescents must assume greater responsibility for managing their condition, including medication adherence and self-monitoring [11]. In addition to psychosocial factors, genetic components play a significant role in the etiology of epilepsy. Advances in genomic technologies have facilitated the identification of genes associated with various epilepsy syndromes, providing insights into disease mechanisms and informing personalized treatment approaches. For example, pharmacogenetic findings from this study highlight the therapeutic implications of specific genetic variants. Patients with *DEPDC5* mutations [12], associated with mTOR pathway dysregulation, may benefit from targeted treatments such as mTOR inhibitors (e.g., everolimus), which have shown promise in related epilepsy syndromes. Similarly, *SLC12A5* mutations [13], affecting K-Cl cotransporter (KCC2) function, suggest a potential role for therapies aimed at restoring chloride homeostasis in neurons, such as bumetanide, which is under investigation for epilepsy management. Additionally, mutations in *ASH1L* [14] and *SETD1A* [15], involved in chromatin remodeling and histone methylation, indicate the potential for epigenetic therapies that modulate gene expression. These examples underscore the importance of integrating pharmacogenetics into clinical practice, enabling precision medicine approaches tailored to the genetic profiles of AOE patients. Expanding research on these therapeutic strategies could lead to improved seizure control and better overall outcomes for patients [16]. However, the interplay between genetic predispositions and psychosocial factors can influence the presentation and progression of epilepsy in adolescents [17]. Understanding both the genetic and psychosocial dimensions of AOE is essential for developing holistic management strategies that address not only seizure control but also the overall well-being of adolescents. This comprehensive approach can improve treatment adherence, enhance quality of life, and support adolescents in navigating the challenges associated with their condition [18].

In this study, we investigated the clinical characteristics of AOE in a cohort of 76 Korean patients, with a particular focus on both genetic factors and psychosocial aspects. We performed genetic testing to identify variants and expand the spectrum of mutations associated with AOE. Additionally, we explored the psychosocial implications of epilepsy in adolescents, aiming to provide a more complete understanding of the condition that can inform clinical practice and support services.

## 2. Materials and Methods

### 2.1. Patients

The cohort comprised 76 patients with AOE treated in the Department of Pediatric Neurology at Daejeon St. Mary’s Hospital (Daejeon, Republic of Korea) between January 2019 and December 2023. The inclusion criteria were as follows: (1) two or more unprovoked seizures; (2) ages between 12 and 18 years (in accordance with Korean legal regulations); and (3) a minimum follow-up period of 36 months after the initiation of antiseizure medication (ASM) treatment. Patients were excluded if they had a history of epilepsy treatment prior to the study period, insufficient clinical data, or had not initiated ASM treatment after their diagnosis during the study period. Seizure types at presentation and epilepsy syndromes were defined according to the International League Against Epilepsy (ILAE) classification. Neurodevelopmental delays (NDDs) were characterized as disabilities primarily affecting the neurological system, including autism spectrum disorder (ASD), intellectual disability (ID), learning disabilities, and cerebral palsy. All patients underwent intelligence quotient (IQ) testing and the Child Behavior Checklist, with the group showing no abnormalities classified as normal. ID was defined as an IQ of 70 or below, accompanied by deficits in at least two areas of adaptive functioning, with onset before age 18. Other NDD were diagnosed based on the criteria specified in the Diagnostic and Statistical Manual of Mental Disorders, Fifth Edition (DSM-5). Data were retrospectively collected by reviewing electronic medical charts, including details on sex, age, seizure type, comorbidities, epilepsy syndrome type, age at onset, electroencephalogram (EEG) results, ASM, recurrence rates, brain magnetic resonance imaging (MRI) findings, and genetic test results.

### 2.2. DNA Extraction, Library Preparation, and Clinical Exome Sequencing

Genomic DNA was isolated from peripheral blood samples using the QIAamp DNA Mini Kit, following the manufacturer’s instructions (Qiagen, Hilden, Germany). Comprehensive singleton clinical exome sequencing (CES) was performed on the proband in each family using a customized Celemics G-Mendeliome Clinical Exome Sequencing Panel (Celemics, Inc., Seoul, Republic of Korea) after routine karyotyping and chromosomal microarray (CMA). The CES was conducted at a CAP-accredited clinical laboratory, GC Genome (Yongin, Republic of Korea), for research purposes. This panel encompasses a wide range of 7000 genes linked to clinically significant Mendelian genetic disorders, covering all clinically relevant regions. In summary, target enrichment was performed using custom-designed RNA oligonucleotide probes in combination with a target enrichment kit (Celemics, Inc.). The pooled libraries were subsequently sequenced in parallel utilizing the DNBSEQ-G400RS High-throughput Sequencing Set on the DNBSEQ-G400 sequencer (MGI Tech Co., Ltd., Shenzhen, China).

### 2.3. Bioinformatic and Segregation Analysis

Sequencing and bioinformatics analyses adhered to the Genome Analysis Tool Kit (GATK) best practices pipeline workflow (https://gatk.broadinstitute.org/hc/en-us; accessed on 13 January 2024). This comprehensive process included base-calling, alignment, variant calling, annotation, and quality control reporting. Sequences were aligned to the hg19 reference genome using BWA-aln, and single nucleotide variants (SNVs) and small insertions or deletions (indels) were identified and validated using GATK version 3.8.0 with HaplotypeCaller, as well as VarScan version 2.4.0. Variant interpretation followed the five-tier classification system recommended by the American College of Medical Genetics and Genomics (ACMG) and the Association for Molecular Pathology (AMP) [19]. Computational tools for assessing missense variant pathogenicity and ClinGen recommendations for PP3/BP4 criteria [20] were also applied. The filtering criteria for identifying potentially pathogenic variants included the following: (1) variants with an allele frequency of less than 0.01; (2) variants located near or within exons of protein-coding genes associated with epilepsy and their clinical significance; (3) de novo or rare variants identified in the proband that are heterozygous, compound heterozygous, or homozygous in the same gene; (4) variants that result in nonsynonymous or nonsense codon changes, affect highly conserved splice sites, or cause frameshift mutations. Variant frequencies in the general population were evaluated using the Genome Aggregation Database (gnomAD) (https://gnomad.broadinstitute.org/; accessed on 1 February 2024). The pathogenicity of candidate causative variants was predicted using five in-silico prediction algorithms: (1) The BayesDel addAF using VarSome (https://varsome.com/; accessed on 1 February 2024) is a deleteriousness prediction meta-score that includes MaxAF and is used for SNVs and indels. The score range in dbNSFP is from −1.11707 to 0.750927, with higher scores indicating a greater likelihood of the variant being pathogenic. A cutoff of 0.0692655 is suggested to distinguish between deleterious (“D”) and tolerated (“T”) variants. (2) The MutationTaster (https://www.mutationtaster.org/; accessed on 10 February 2024) was employed. (3) VEST-4 (Variant Effect Scoring Tool-4) (https://www.cravat.us/CRAVAT/; accessed on 10 February 2024) is a machine learning-based method used to predict the functional significance of non-silent variants by estimating their likelihood of being pathogenic. The VEST score, specifically designed for missense germline variants, ranges from 0 (indicating likely benign) to 1 (indicating likely pathogenic). Additionally, the VEST *p*-value provides an empirical measure of the probability that a benign variant is incorrectly classified as pathogenic. (4) phastCons scores (ranging from 0 to 1) estimate the probability that a nucleotide belongs to a conserved element. These scores are derived from the multiple alignment of genome sequences across 46 species. Higher scores (closer to 1) indicate a stronger likelihood of conservation. Unlike phyloP, phastCons takes into account both the individual nucleotide position and its surrounding alignment context. (5) phyloP scores (ranging from −14 to +6) evaluate conservation at individual genomic positions independently of neighboring columns. These scores measure evolutionary rates, identifying conservation (slower evolution than expected under neutral drift) with positive values and acceleration (faster evolution) with negative values. Conserved sites are assigned positive scores, whereas fast-evolving sites receive negative scores. The presence of candidate causative variants classified as likely pathogenic variant (LPV), pathogenic variant (PV), or variant of uncertain significance (VUS) associated to epilepsy in the proband was confirmed by bidirectional Sanger sequencing using a 3730xl DNA Analyzer (Applied Biosystems, Foster City, CA, USA). To determine the origin of these rare variants, Sanger sequencing was also performed on the patient’s parents.

### 2.4. Statistical Analysis

The clinical and genetic characteristics of AOE with or without NDD were compared using the Student’s *t*-test for continuous factors and Fisher’s exact test for categorical factors. All statistical analyses were performed using MedCalc statistical software (version 19.8.3, MedCalc Software, Ltd., Ostend, Belgium). A *p*-value of less than 0.05 was considered statistically significant.

## 3. Results

**Figure 1:**
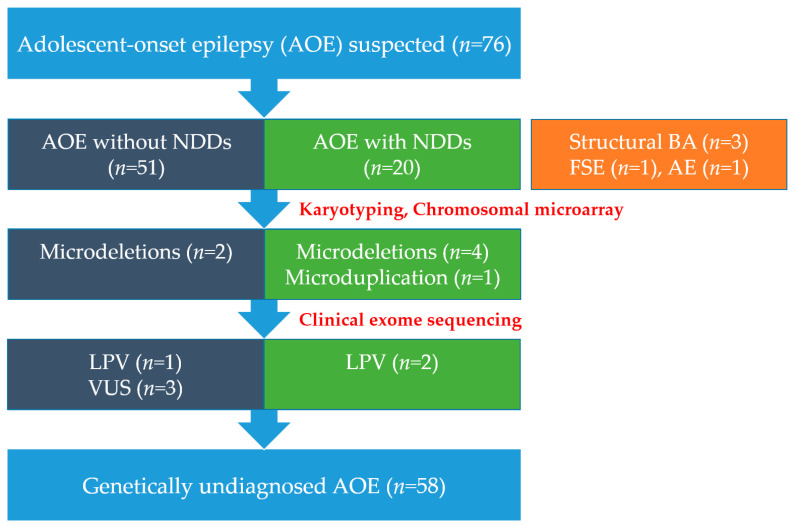
Diagnostic workflow for genetic diagnosis in adolescent-onset epilepsy. NDD, neurodevelopmental delay; BA, brain abnormalities; FSE, febrile status epilepticus; AE, autoimmune encephalitis; LPV, likely pathogenic variant; VUS, variant of uncertain significance.

Of seventy-six patients, 71 met all inclusion criteria and five who did not meet the criteria were excluded: three structural brain abnormalities, one febrile status epilepticus, and one autoimmune encephalitis. Of these, 37 (52%) were male, and 34 (48%) were female. The mean age at seizure onset was 14.6 years. The most common type of seizure, based on eyewitness accounts, was generalized tonic-clonic seizures (GTCA), which accounted for 76% (54 patients). According to the ILAE classification of epilepsy syndromes, 58 patients (82%) were classified as having idiopathic generalized epilepsy (IGE). Within this group, one patient (1%) had juvenile absence epilepsy (JAE), four patients (6%) had juvenile myoclonic epilepsy (JME), and 53 patients (75%) had epilepsy with GTCA (EGTCA). Thirteen patients (18%) had focal epilepsy, including temporal lobe epilepsy (two patients, 15%) and focal epilepsy of unknown cause (eleven patients, 85%). EEG results were abnormal in 56 patients (79%), with the most commonly observed abnormalities being generalized spikes and slow waves, consistent GTCA, and focal epileptiform discharges associated with focal epilepsy. Other abnormalities included paroxysmal sharp waves and high-voltage wave complexes, reflecting the diverse range of seizure types and epilepsy syndromes within the cohort. Among the patients, 56 (79%) were treated with a single ASM, while 15 patients (21%) required polytherapy. ASM was discontinued after 2 years of seizure freedom and normalized EEG results. However, seizures recurred in 16 patients (23%) after discontinuation of ASM. Additionally, 10 patients (14%) had a history of prior febrile seizures, and 18 patients (25%) had a family history of epilepsy. To better understand the genetic risk factors, inheritance patterns within affected families were further explored. Among these families, specific genetic variants, such as those in *DEPDC5*, *SLC12A5*, and *ASH1L*, were identified, supporting a potential autosomal dominant inheritance pattern with variable expressivity. For example, patient n1yj and her father exhibited similar seizure onset ages and carried the same *ASH1L* mutation, while patient n5sy and her mother shared an *SLC12A5* mutation with age-related variability in clinical expression. These findings highlight the significance of genetic testing in familial cases to elucidate inheritance patterns, identify susceptibility genes, and inform personalized treatment strategies. In the AOE without NDDs, 10 patients (20%) were able to safely discontinue ASMs, while 4 patients (20%) in the NDD group also discontinued them, with no statistically significant difference observed between the groups. Furthermore, a sequential algorithmic approach to genetic testing was applied to AOE patients to enhance understanding of its underlying causes and improve diagnostic accuracy (Figure 1).

### 3.1. Presentation of Case Series in Adolescent-Onset Epilepsy Without Neurodevelopmental Delays

#### 3.1.1. Patient n1yj with *ASH1L* c.3916C>T/p.(Arg1306Ter) Variant

**Figure 2:**
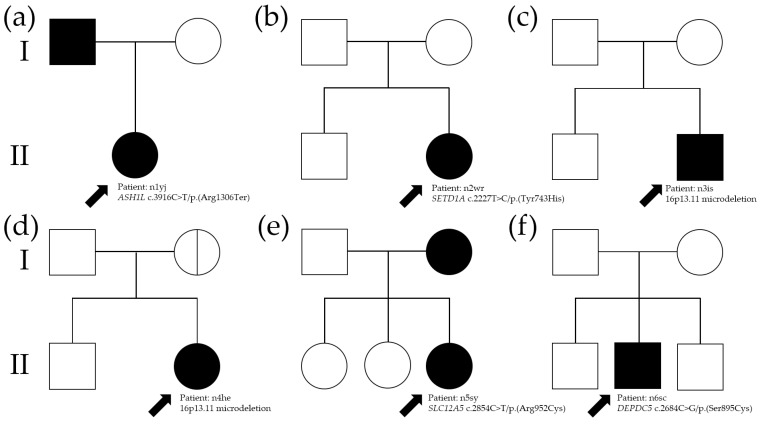
Pedigrees for families with adolescent-onset epilepsy without neurodevelopmental delay, along with the segregation of identified variants within each family. The black arrow indicates the proband in each family. (**a**) A family carrying *ASH1L* variant. (**b**) A family carrying *SETD1A* variant. (**c**) A family carrying 16p13.11 microdeletion. (**d**) A family carrying 16p13.11. (**e**) A family carrying *SLC12A5* variant (**f**) A family carrying *DEPDC5* variant. I, first generation; II, second generation.

**Figure 3:**
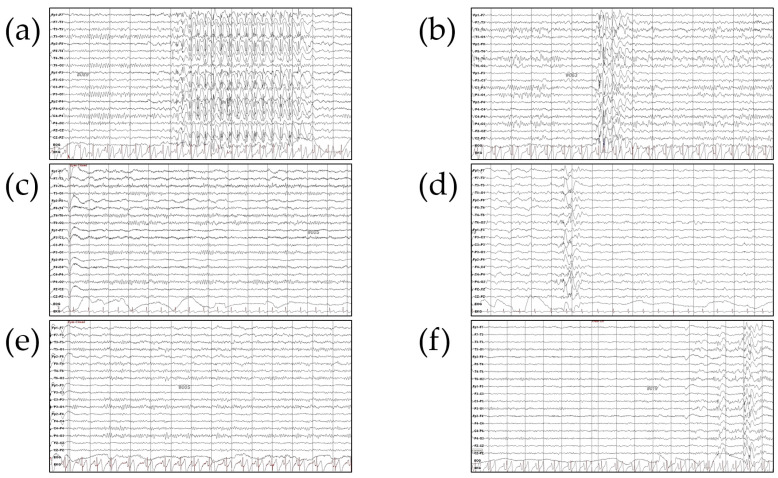
Electroencephalogram (EEG) findings in adolescent-onset epilepsy patients without neurodevelopmental delays, carrying genetic alterations. The interictal EEG recordings showed the following: (**a**,**b**) Generalized spikes and slow waves in patient n1yj and patient n2wr; (**c**) a normal background with no epileptiform discharges in patient n3is; (**d**) paroxysmal generalized sharp waves in patient n4he; (**e**) normal interictal waves in patient n5sy; (**f**) high-voltage sharp and wave complexes in the right temporal areas, occasionally spreading more generally, in patient n6sc.

She (II-1 in Figure 2a) was a 13-year-old female, born after an uneventful pregnancy and delivery, with normal developmental milestones and a low-normal IQ of 80 (9th percentile) at age 13. She exhibited no behavioral problems but experienced learning difficulties. Her father, who began experiencing seizures at age 14, also had a borderline IQ of 81 (10th percentile). He is currently being treated with levetiracetam and clonazepam. At age 13, she experienced three GTCAs over three months. Her neuroimaging results were normal, and interictal EEG revealed generalized spikes and slow waves (Figure 3a). She was treated with valproic acid and levetiracetam, achieving seizure freedom. She graduated from an agricultural high school and now works as a farmer.

#### 3.1.2. Patient n2wr with *SETD1A* c.2227T>C/p.(Tyr743His) Variant

She (II-2 in Figure 2b) was a 14-year-old female, the second child of non-consanguineous parents, with a normal developmental history and an IQ of 100 (50th percentile). There was no reported family history of neurological diseases. She developed GTCA at age 14. Her cranial MRI was normal, and the interictal EEG revealed generalized spikes and slow waves (Figure 3b). She was initially treated with levetiracetam, achieving seizure control. ASM was discontinued after 2 years, but seizures recurred, and she was successfully managed with levetiracetam and lamotrigine. She now works as a computer technician after graduating from high school.

#### 3.1.3. Patient n2wr n3is with 16p13.11 Microdeletion

He (II-2 in Figure 2c) was a 15-year-old male, born to non-consanguineous healthy parents, with normal development and intelligence. There was no family history of seizures. He began experiencing GTCA at age 15. His brain MRI was normal, and the background EEG showed no epileptiform discharges (Figure 3c). He was treated with levetiracetam for 2 years, but seizures recurred after discontinuation. He resumed levetiracetam and has remained seizure-free since. He graduated from a vocational high school specializing in industry and now works as a licensed electrician.

#### 3.1.4. Patient n4he with 16p13.11 Microdeletion

She (II-2 in Figure 2d) was a 14-year-old female, born at full term to non-consanguineous healthy parents, with age-appropriate development and an IQ of 109 (72nd percentile). Her mother experienced a single episode of fever-induced seizures at age 10 due to acute pharyngotonsillitis but has not had any subsequent seizures. The proband’s mother graduated from college, works as an office employee, and remains seizure-free. She developed paroxysmal GTCA at age 14. Her brain MRI was normal, and the interictal EEG showed paroxysmal generalized sharp waves (Figure 3d). She achieved seizure control with levetiracetam.

#### 3.1.5. Patient n2wr with *SLC12A5* c.2854C>T/p.(Arg952Cys) Variant

She (II-3 in Figure 2e) was a 14-year-old female, born at full term without complications and with no significant medical issues. She is currently studying art at university. Her mother experienced epilepsy starting at age 22, was treated with phenytoin until age 29, and has remained seizure-free since. She experienced GTCA at age 14. Her brain MRI was normal, and the interictal EEG showed normal waves (Figure 3e). She was treated with levetiracetam, achieving seizure freedom for 2 years. She has managed her daily life independently.

#### 3.1.6. Patient n2wr with *DEPDC5* c.2684C>G/p.(Ser895Cys) Variant

He (II-2 in Figure 2f) was a 15-year-old male, the second child of non-consanguineous Korean parents, with normal development. He has no family history of epilepsy or neurological disorders. He began experiencing paroxysmal episodes of unconsciousness with arm stiffness, preceded by a sensation of discomfort, at age 15. His brain MRI was normal, and the interictal EEG revealed high-voltage sharp and wave complexes in the right temporal areas (Figure 3f). He was treated with valproic acid, achieving complete seizure control and normalization of EEG findings. He is currently a high school baseball player and plans to major in physical education at university.

### 3.2. Genetic Characterization of Patients with Adolescent-Onset Epilepsy

Genetic testing identified a spectrum of specific mutations and structural variations associated with epilepsy, providing critical insights into the underlying disease mechanisms and their implications for diagnosis and treatment. In this study, a total of six genetic alterations, including four LPVs or VUS and two copy number variations (CNVs), were detected in patients with AOE without NDD. In contrast, patients with AOE accompanied by NDD exhibited a broader genetic spectrum, including two LPVs, five CNVs, and two additional variants of interest. The clinical and genetic characterization of patients with AOE (six AOE without NDD; seven AOE with NDD) due to genetic alterations is summarized in Table 1.

Additionally, the in silico analysis results of candidate genetic alterations in patients with AOE are detailed in Table 2.

### 3.3. Comparison of Adolescent-Onset Epilepsy Based on the Presence of Neurodevelopmental Delay

Clinical manifestations in patients with AOE were compared based on the presence and specific subtypes of NDD, such ASD, ID, and learning disabilities, to identify the unique genetic and clinical features of AOE. Patients with abnormalities on brain MRI or epilepsy related to infections, immune factors, or metabolic disorders were excluded. As a result, a statistically significant difference was observed in the prevalence of males and the yield of genetic tests between AOE with and without NDD. Patients with AOE without NDD had a lower prevalence of males (49%) compared to those with AOE with NDD (60%) (*p* = 0.007). However, AOE with NDD carried more genetic alterations (35%) compared to AOE without NDD (12%) (*p* = 0.011). Among patients with NDD, those with ID showed the highest frequency of genetic findings (e.g., CNVs), followed by ASD and combined ID-ASD cases. This stratification underscores the need for tailored genetic investigations to elucidate the underlying molecular mechanisms in specific NDD subtypes. Other factors, such as age, seizure type, epilepsy syndrome, recurrence rate, family history of epilepsy, and prior febrile seizures did not show statistically significant differences (Table 3). This detailed genetic analysis of AOE highlights the heterogeneity of epilepsy and the critical role of genetics in understanding its pathophysiology and tailoring interventions.

## 4. Discussion

AOE, classified into three syndromes, namely, JAE, JME, and EGTCA, typically begins in childhood or adolescence and is often associated with normal development and intellect. Recent research by Cerulli Irelli et al., involving female patients of childbearing age with IGE, demonstrated that treatment with levetiracetam or lamotrigine effectively reduced seizure frequency [21,22]. In this study, 82% of the cohort was diagnosed with IGE, with EGTCA being the most common syndrome (75%). This finding aligns with studies on both adolescent-onset and childhood-onset epilepsies, where the percentage of patients with IGE ranged from 43.9% to 60% [23,24,25]. The low prevalence of focal epilepsy in this cohort, due to the rarity of symptomatic etiologies, may explain the higher occurrence of generalized epilepsy. A Canadian study reported that approximately 85% of cases achieved seizure control with monotherapy [5]. Comorbid conditions such as NDD were noted in 28% of patients. The proportions of monotherapy (79%) and combined NDD (28%) are consistent with previous reports.

Our study reports a higher yield of genetic abnormalities in patients with AOE and NDDs (35%) compared to those without NDDs (12%) (*p* = 0.011). This significant finding underscores the potential genetic underpinnings associated with neurodevelopmental impairments in epilepsy. However, further exploration is needed to understand the mechanisms driving these differences. Future studies should focus on investigating the genetic pathways and environmental interactions that contribute to the increased prevalence of genetic abnormalities in AOE with NDD, which could provide deeper insights into the etiology and inform more targeted interventions. In this study, we observed a familial component in 25% of patients with AOE, suggesting a notable genetic influence. Among these cases, specific genetic alterations were identified that provide insights into inheritance patterns. For instance, patient n1yj and her father (I-1 in Figure 2a) both carried a missense variant in *ASH1L* (c.3916C>T/p.(Arg1306Ter)). This variant, coupled with their borderline IQ scores and similar seizure onset ages, highlights a potential autosomal dominant pattern of inheritance with variable expressivity. Similarly, in patient n5sy, both she and her mother (I-2 in Figure 2e) carried a missense variant in *SLC12A5* (c.2854C>T/p.(Arg952Cys)). While the proband exhibited seizure onset at age 14, her mother developed epilepsy later, at age 22, which resolved following treatment. This suggests incomplete penetrance and age-related variability in clinical expression for *SLC12A5* variants. In patient n6sc, a variant in *DEPDC5* (c.2684C>G/p.(Ser895Cys)) was identified. This gene is well-established in familial focal epilepsy with variable foci, and while no family members were clinically affected in this case, the finding aligns with the known reduced penetrance observed in *DEPDC5*-associated epilepsies. Overall, familial cases in this cohort were more likely to exhibit variants in genes associated with established epilepsy syndromes, such as *ASH1L*, *SLC12A5*, and *DEPDC5*. These examples illustrate the importance of evaluating family histories and performing genetic testing to uncover potential inheritance patterns and inform clinical management. Our study provides a detailed breakdown of the cohort’s characteristics, including sex, age at onset, seizure type, epilepsy syndrome, and recurrence rates. However, potential impacts of environmental or lifestyle factors on AOE have not been explored. Incorporating data on these variables, such as dietary habits, physical activity, sleep patterns, stress levels, or exposure to environmental toxins, could enhance the understanding of non-genetic contributors to AOE. Future research should integrate these factors to offer a more comprehensive view of the condition and its multifaceted etiology.

In this study, several genetic alterations were identified in patients with AOE without NDD. *ASH1L* (ASH1 like histone lysine methyltransferase, OMIM *607999) encodes a histone methyltransferase crucial for epigenetic modifications. Loss-of-function (LoF) mutations in *ASH1L* impair H3K36 methylation and can lead to developmental disorders [26,27]. While the precise pathogenic mechanisms of *ASH1L* mutations are not fully understood, truncating variants are generally considered LoF, whereas missense variants might act in a dominant-negative or gain-of-function manner [28,29,30]. Although the exact pathogenic mechanisms of *ASH1L* mutations are not fully understood, truncating variants are generally considered LoF, while missense variants might act in a dominant-negative or gain-of-function manner [31]. Additionally, ASH1L expression and levels of H3K4me3 are significantly reduced in the prefrontal cortex (PFC) of postmortem tissues [32]. Deficiency of Ash1l in the PFC results in reduced GABAergic inhibition, increased glutamate transmission, and heightened excitability of PFC pyramidal neurons, which are associated with seizures [33]. About 7% of patients with *ASH1L* mutations have been reported to experience seizures, primarily during early childhood [30]. In this study, patient n1yj carried a likely pathogenic missense variant, p.(Tyr746His), in *ASH1L*, indicating that *ASH1L* may be a novel susceptibility gene for AOE with neurodevelopmental impairment. Recently, 55% of patients with pathogenic *ASH1L* variants experienced seizures, including individuals with borderline intelligence; both the proband and her father exhibited borderline intelligence as well. Animal studies have elucidated mechanisms of seizures related to gene expression in the brain. While it is still premature to definitively identify causative genes, we have described a candidate or susceptibility gene in this family. The presence of a novel nonsense mutation further supports the possibility of a susceptibility gene [14].

Post-translational modifications of histones and their impact on gene regulation have become increasingly significant in various neurological disorders. *SETD1A* (SET Domain Containing 1A) is a chromatin remodeler that regulates gene expression by modifying mono-, di-, and trimethylation marks on Histone H3-Lysine-4 (H3K4me1/2/3) [34]. H3K4 methylation is primarily associated with transcriptional activation, with different methylated forms found at promoters or enhancers, which are crucial for preventing DNA damage at stalled replication forks [35]. Reports on *SETD1A*-related epilepsy are limited, with clinical features observed in approximately 23% of cases [15,36,37,38,39]. Among the seven reviewed patients, four had missense variants, three had frameshift mutations, and six had de novo mutations. Most patients experienced seizure onset during infancy, primarily presenting with tonic-clonic seizures. Effective treatment strategies for *SETD1A*-related epilepsy remain unclear. The *SETD1A* variant (c.2227T>C/p.Tyr743His) may predispose individuals to AOE with neurodevelopmental impairment (NI), thereby expanding the mutational and phenotypic spectrum associated with SETD1A. This case highlights novel clinical phenotypes and enhances our understanding of SETD1A-related epilepsy compared to previous reports.

The *SLC12A5* (Solute carrier family 12 member 5) gene encodes the neuronal KCC2 channel, which plays a crucial role in extruding intracellular chloride from mature neurons [13]. When chloride levels within neurons are low, GABA and glycine binding to their ionotropic receptors induces chloride influx, resulting in hyperpolarization and subsequent neuronal inhibition [40]. *SLC12A5* is exclusively expressed in the central nervous system. Kahle et al. identified two heterozygous missense variants in *SLC12A5*—p.Arg952His and p.Arg1049Cys—that were notably enriched among individuals of French Canadian origin with IGE type 14, compared to controls [41]. The p.Arg952His variant was found in 5 of 380 patients with IGE (allele frequency of 0.66%) and in 5 of 1214 controls (allele frequency of 0.21%), resulting in an odds ratio of 3.21 for developing EIG (*p* = 0.065). Additionally, Puskarjov et al. identified the heterozygous p.Arg952His variant in the *SLC12A5* gene in three members of an Australian family with febrile seizures [42]. Patients (II-3 and I-2 in Figure 2e) carried the *SLC12A5* mutation (c.2854C>T/p. Arg952Cys), affecting the same amino acid position. These findings suggest that impaired KCC2 function, caused by genetic variants, may increase susceptibility to epilepsy, specifically IGE (OMIM #616685).

The *DEPDC5* (Dep Domain-Containing Protein 5) gene encodes a protein integral to the GATOR1 complex, which inhibits the mTOR (mammalian target of rapamycin) signaling pathway responsible for regulating cell growth and proliferation [43]. Dysregulation of DEPDC5 leads to activation of the mTORC1 pathway, resulting in abnormal neuronal morphology, disrupted cortical laminar structure, cortical neuronal hyperexcitability, and clinical seizures [44]. *DEPDC5*-related epilepsy is characterized by focal seizures and includes conditions such as familial focal epilepsy with variable foci (OMIM #620504) and autosomal dominant sleep-related hypermotor epilepsy [45]. Other reported seizure types associated with *DEPDC5* mutations include mesial temporal lobe epilepsy, autosomal dominant epilepsy with auditory features, infantile spasms, and developmental and epileptic encephalopathy (OMIM #604364) [46]. Most patients with *DEPDC5* mutations have a normal brain MRI; however, some may exhibit epilepsy associated with cortical malformations or neuropsychiatric disorders such as developmental delay/ID and ASD [45]. Interictal EEG findings typically show focal epileptiform abnormalities with a normal background rhythm [47]. Although the average age of onset for *DEPDC5*-related epilepsy is during the preschool years [48,49], seizures in this study began at 15 years old. While 50% of patients with *DEPDC5* mutations experience refractory epilepsy, 80% of those with refractory epilepsy achieve seizure freedom or significant improvement following surgical resection [50,51]. Asymptomatic heterozygotes are common in *DEPDC5*-related epilepsy families, leading to reduced penetrance of approximately 60% [12,52]. Further research is needed to clarify the functional impacts of different *DEPDC5* variants, understand its role in epileptogenesis, and develop targeted therapies.

On the other hand, CNVs play a significant role in the genetic risk and etiology of both rare and common epilepsies. Studies have identified pathogenic CNVs in approximately 10–13% of individuals with epilepsy who also have ID or ASD. While these microdeletions can also be present in unaffected individuals at low frequencies, they are considered risk alleles for epilepsy, with odds ratios ranging from 5 to 68 [53,54]. Recurrent microdeletions, such as those at 15q11.2, 15q13.3, and 16p13.11, are detected in approximately 1% of patients with genetic generalized epilepsies [55,56,57,58]. CNVs contribute significantly to the complex genetic landscape of epilepsy within families, with recurrent CNVs serving as prominent risk factors that exhibit incomplete penetrance. Hannes et al. reported 16p13.11 deletions in patients with ID and epilepsy, though ID is not consistently observed among all epilepsy patients with these deletions [59]. Microdeletions at 16p13.11 have been associated with IGE and other epilepsy disorders [57,59]. Even among individuals with identically sized 16p13.11 deletions, there is considerable variability in epilepsy phenotypes. This variability suggests that 16p13.11 deletions can predispose individuals to AOE with or without neurocognitive impairments [57,59,60,61]. The *NDE1* gene, located within this region, encodes a protein crucial for the proper positioning of cells during cortical development. However, the mechanisms by which 16p13.11 copy number changes lead to such diverse neuropsychiatric or neurodevelopmental conditions remain unclear.

Seven individuals with AOE accompanied by NDD in this study were found to carry genetic alterations. Epilepsy genes often produce a diverse range of seizure types, severities, intellectual functioning, and comorbidities [62,63]. These genes are also linked to ASD, ID, and schizophrenia, expanding their phenotypic spectrum to include a broader range of NDD [64]. Seizures are common in individuals with NDs, affecting 21.5% of those with ASD and ID, and 8% of those with ASD without ID [65]. In this study, 71% of patients had CNVs, including deletions and duplications at 16p11.2, 12p13.33-p13.32, 2q37, and 15q11.2-q13.1. These CNVs alter genomic segment dosage and are established risk factors for various types of epilepsy [66]. Additionally, mutations in *SLC6A8* (Solute carrier family 6 member 8) and *SMARCE1* (SWI/SNF related BAF chromatin re-modeling complex subunit E1) were identified. SLC6A8 deficiency, an X-linked disorder, causes cerebral creatine deficiency syndrome, which is associated with ID, epilepsy, and behavioral disorders [67,68]. Coffin–Siris syndrome type 5, caused by heterozygous mutations in the *SMARCE1* gene on chromosome 17q21, is characterized by delayed psychomotor development, ID, dysmorphic features, and seizures [69,70,71].

Identifying a pathogenic variant requires a thorough evaluation of multiple lines of evidence. This includes assessing the frequency of the variant, or similar variants in the same gene, among control participants of comparable ethnicity. It also involves examining the segregation of the variant with affected status within families and, when possible, determining its functional impact at the protein, cellular, or model organism levels [19]. Additionally, other genetic mechanisms such as modifier genes, nucleotide repeats, and epigenetic factors likely play a role [72]. Both genetic and environmental factors contribute to the risk of developing epilepsy, with incomplete penetrance and variable expressivity stemming from these influences [73]. Comprehensive phenotyping of patients after identifying a mutation is crucial for understanding the full range of phenotypic manifestations associated with that gene mutation, helping to minimize phenotypic bias. Expanding our understanding of the phenotypic spectrum and the contributions of both rare and common variants challenges the epilepsy research and clinical communities to shift from a deterministic view of genetic changes to a more nuanced understanding of how these changes contribute to disease risk within complex neurobiological systems. More than 30 different mutated genes have been identified in families affected by rare autosomal dominant monogenic epilepsies with high penetrance [74,75]. Initially, these mutations were predominantly found in genes coding for ion channels. However, mutations have also been discovered in genes unrelated to ion channels, including those for neuronal receptors, transcription factors, and enzymes. Familial monogenic epilepsies represent a small proportion (5–10%) of all genetic epilepsies [76,77]. The genetic basis of more common epilepsies is likely multifactorial, involving contributions from multiple genes that may act within an individual or vary across different individuals with the same syndrome.

This study has several limitations. In this study, we provided detailed descriptions of six representative cases from a cohort of 51 patients with AOE without NDD. These cases were selected based on their genetic findings and clinical diversity to highlight the spectrum of AOE presentations within this subgroup. While these individual case studies offer valuable insights into genetic associations, treatment responses, and clinical outcomes, we acknowledge that the small sample size is insufficient to capture meaningful patterns or trends across the entire population. This limitation stems from the study’s design, which aimed to illustrate distinct genetic and clinical features rather than perform a comprehensive statistical analysis of all patients. The decision to focus on these six cases was made to balance the depth of analysis with the need to provide detailed phenotypic and genetic data. However, the limited scope may hinder the generalizability of the findings to the broader cohort of AOE patients without NDD. To address this gap, future studies should aim to include systematic and detailed analyses of the full cohort. This would allow for identifying statistically significant patterns, trends, and correlations across a larger sample size. Additionally, comparative analyses between patients with and without NDD would provide a more comprehensive understanding of the heterogeneity in AOE presentations and outcomes. Such approaches could also facilitate the development of more targeted diagnostic and treatment strategies for the diverse spectrum of AOE.

While this study aims to identify variants and expand the spectrum of mutations associated with AOEs, its findings are primarily based on individual genetic mutations identified in specific patients with AOE, as shown in Table 1. However, the study does not conduct a large-scale analysis of these mutations or establish comprehensive links between the mutations and the pathophysiology of AOE. Our study employs CMA and CES to identify genetic abnormalities, providing a robust and targeted approach for genetic diagnosis. However, these methods have inherent limitations, as CES focuses primarily on exonic regions and CMA detects larger CNVs. Integrating other approaches, such as whole genome sequencing, could capture additional variants, including those in non-coding regions, structural variants, and small indels, which may contribute to the genetic etiology of AOE. Future studies should consider combining CES and CMA with WGS to provide a more comprehensive genetic landscape of AOE. This limitation highlights the need for further investigation into the broader implications of these findings. Future research should include larger cohorts and in-depth functional studies to validate the pathogenicity of these variants and explore their potential roles in the development and clinical manifestations of AOE. Emphasizing this need for additional research in the discussion section could strengthen the study’s contribution to the field. Through genetic testing of AOE patients, causative gene variants and CNVs were identified. In the AOE without NDD group, susceptibility gene variants potentially associated with epilepsy were also described. A limitation of this study is that the genetic testing was conducted using blood samples, meaning variants expressed only in brain tissue or somatic mosaicism could not be detected.

## 5. Conclusions

In conclusion, we investigated the characteristics of AOE and identified genetic variants associated with the condition in a cohort of 76 Korean patients. By employing CES and CMA, we identified a range of genetic alterations, including specific pathogenic and likely pathogenic variants, as well as CNVs. Notably, we observed differences in the prevalence and type of genetic findings between AOE patients with and without NDD, highlighting the genetic heterogeneity of AOE. Our findings emphasize the potential of CES and CMA as effective tools for uncovering genetic factors underlying AOE, particularly in cases with complex etiologies or locus heterogeneity. Specific variants identified, such as those in *ASH1L*, *SETD1A*, *DEPDC5*, and *SLC12A5*, expand the spectrum of known genetic contributions to AOE and suggest potential links between these mutations and clinical presentations. This study also highlighted that genetic variants were more prevalent in patients with NDD, whereas patients without NDD demonstrated milder clinical features. These results underscore the importance of integrating genetic testing into the diagnostic workflow for AOE to enhance understanding of its molecular underpinnings and guide personalized treatment approaches. Future studies should build on these findings by exploring genotype–phenotype correlations, evaluating the functional implications of identified variants, and expanding the sample size to validate the observed patterns.

## Figures and Tables

**Table 1:** Clinical and genetic characterization of patients with adolescent-onset epilepsy due to genetic alterations.

Pt	S/A	Seizure Type	Epilepsy Syndrome	EEG	NDD	Genetic Alteration	OMIM Phenotype	Origin
n1yj	F/13	generalized	EGTCA	pos	neg	NM_018489.3(*ASH1L*): c.3916C>T/p.(Arg1306Ter)	MRD52 (# 617796)	paternal
n2wr	F/14	generalized	EGTCA	pos	neg	NM_014712.3(*SETD1A*): c.2227T>C/p.(Tyr743His)	EPEO2 (# 618832)	sporadic
n3is	M/15	generalized	EGTCA	neg	neg	16p13.11 microdeletion	n.a.	sporadic
n4he	F/15	generalized	EGTCA	pos	neg	16p13.11 microdeletion	n.a.	maternal
n5sy	F/14	generalized	EGTCA	neg	neg	NM_020708.5(*SLC12A5*): c.2854C>T/p.(Arg952Cys)	EIG14 (# 616685)	maternal
n6sc	M/14	focal	FE	pos	neg	NM_001364318.2(*DEPDC5*): c.2684C>G/p.(Ser895Cys)	FFEVF1 (# 604364)	sporadic
p1rn	F/14	focal	FE	pos	pos	15q11.2q13.1 deletion	PWS (# 176270)	sporadic
p2hy	F/14	generalized	EGTCA	pos	pos	NM_005629.4(*SLC6A8*): c.1222_1224del/p.(Phe408del)	CCDS1 (# 300352)	sporadic
p3sw	M/18	focal	FE	pos	pos	16p11.2 deletion	16p11.2 deletion (# 611913)	sporadic
p4yc	M/14	generalized	EGTCA	pos	pos	2q37 deletion	2q37 deletion (# 600430)	sporadic
p5ny	F/12	generalized	EGTCA	pos	pos	12p13.33p13.32 deletion	n.a.	maternal
p6rw	F/14	generalized	EGTCA	pos	pos	NM_003079.5(*SMARCE1*): c.181A>G/p.(Lys61Glu)	CSS5 (# 616938)	sporadic
p7sj	F/14	generalized	EGTCA	pos	pos	16p11.2 duplication	16p11.2 dup (# 614671)	maternal

Pt, patient; S/A, sex/age at diagnosis; NDD, neurodevelopmental delay; neg, negative; pos, positive; FHx, family history; OMIM, Online Mendelian Inheritance in Man; EGTCA, epilepsy with generalized tonic-clonic seizure alone; FE: focal epilepsy; MRD52, intellectual developmental disorder, autosomal dominant 52; EPEO2, epilepsy, early-onset, 2, with or without developmental delay; EIG14, epilepsy, idiopathic generalized, susceptibility to, 14; FFEVF1, epilepsy, familial focal, with variable foci 1; PWS, Prader–Willi syndrome; CCDS1, cerebral creatine deficiency syndrome 1; CSS5, Coffin–Siris syndrome 5; 16p11.2 dup, 16p11.2 duplication.

**Table 2:** In silico analysis results of candidate genetic alterations in patients with adolescent-onset epilepsy.

Case	Genetic Alteration	Class (ACMG)	gnomAD *	BayesDel addAF	MuTest	VEST-4 (*p* Value)	PhyloP	PhCon
n1yj	NM_018489.3(*ASH1L*): c.3916C>T/p.(Arg1306Ter)	LPV (PVS1)	n.f.	Pathogenic Strong	DisCau	0.83 (0.00173)	1.422	1
n2wr	NM_014712.3(*SETD1A*): c.2227T>C/p.(Tyr743His)	VUS (PM2)	n.f.	Pathogenic Moderate	polym	0.563 (0.14837)	1.012	0.714
n3is	16p13.11 microdeletion	PV (PVS1)	n.f.	n.a.	n.a.	n.a.	n.a.	n.a.
n4he	16p13.11 microdeletion	PV (PVS1)	n.f.	n.a.	n.a.	n.a.	n.a.	n.a.
n5sy	NM_020708.5(*SLC12A5*): c.2854C>T/p.(Arg952Cys)	VUS (PM4)	0.00001193	Benign Moderate	DisCau	0.637 (0.10626)	2.8	1
n6sc	NM_001364318.2(*DEPDC5*): c.2684C>G/p.(Ser895Cys)	VUS (PM2)	n.f.	Uncertain	DisCau	0.622 (0.11355)	4.389	1
p1rn	15q11.2q13.1 deletion	PV (PVS1)	n.f.	n.d.	n.d.	n.d.	n.d.	n.d.
p2hy	NM_005629.4(*SLC6A8*): c.1222_1224del/p.(Phe408del)	PV (PVS1)	0.00004603	Pathogenic Strong	DisCau	0.88 (0.00105)	1.613	1
p3sw	16p11.2 deletion	PV (PVS1)	n.f.	n.d.	n.d.	n.d.	n.d.	n.d.
p4yc	2q37 deletion	PV (PVS1)	n.f.	n.d.	n.d.	n.d.	n.d.	n.d.
p5ny	12p13.33p13.32 deletion	PV (PVS1)	n.f.	n.d.	n.d.	n.d.	n.d.	n.d.
p6rw	NM_003079.5(*SMARCE1*): c.181A>G/p.(Lys61Glu)	LPV (PM2)	n.f.	Pathogenic Moderate	DisCau	0.743 (0.02008)	5.211	1
p7sj	16p11.2 duplication	PV (PVS1)	n.f.	n.d.	n.d.	n.d.	n.d.	n.d.

* Allele count was found only in heterozygous individuals. ACMG, American College of Medical Genetics and Genomics classification; gnomAD, genome aggregation database v2.1.1; MuTest, mutationtester; VEST-4, Variant Effect Scoring Tool-4; PhyloP, phylogenetic *p*-values; PhCon, phylogenetic conservation; LPV, likely pathogenic variant; VUS, variant of uncertain significance; PV, pathogenic variant; DisCau, disease causing; polym, polymorphism; n.f., not found; n.d.; not done; n.a. not available.

**Table 3:** Comparison of clinical manifestations in adolescent-onset epilepsy patients based on the presence of neurodevelopmental delays.

Characteristics	71 AOE (100%)	51 AOE Without NDD (72%)	20 AOE with NDD (28%)	*p*-Value
Male, *n*	37 (52%)	25 (49%)	12 (60%)	0.007
Median age, years (range)	14.6 (12–18.9)	14.4 (12–18.5)	15.4 (12.1–18.9)	0.10
Seizure type, *n*				0.26
Focal (±generalization)	17 (24%)	13 (25%)	4 (20%)	
Generalized	54 (76%)	38 (75%)	16 (80%)	
Epilepsy syndrome, *n*				0.44
Focal epilepsy	13 (18%)	9 (18%)	4 (20%)	
JAE	1 (1%)	1 (2%)	0	
JME	4 (6%)	4 (8%)	0	
EGTCA	53 (75%)	37 (73%)	16 (80%)	
Electroencephalogram, *n*				0.31
Abnormal	56 (79%)	41 (80%)	15 (75%)	
Normal	15 (21%)	10 (20%)	5 (25%)	
Antiseizure medications (ASM), *n*				0.45
Monotherapy	56 (79%)	41 (80%)	15 (75%)	
Duotherapy	13 (18%)	8 (16%)	5 (25%)	
Three or more	2 (3%)	2 (4%)	0	
No. of ASM, mean ± standard deviation	1.31	1.33 ± 0.26	1.25 ± 0.19	0.27
Seizure relapse after ASM discontinuation, *n*	16 (23%)	12 (24%)	3 (15%)	0.21
Prior febrile seizures, *n*	10 (14%)	8 (16%)	2 (10%)	0.27
Family history of epilepsy, *n*	18 (25%)	14 (27%)	4 (20%)	0.26
Mean follow-up period, months (range)	40 (36–85)	40 (36–60)	45 (36–85)	0.12
Yield of genetic tests *, *n*	13 (18%)	6 (12%)	7 (35%)	0.011

AOE, adolescent-onset epilepsy; NDD, neurodevelopmental delay; JAE, juvenile absence epilepsy; JME, juvenile myoclonic epilepsy; EGTCA, epilepsy with generalized tonic-clonic seizure alone. * (Likely) pathogenic variants or variants of uncertain significance were included in the comparison of genetic test yields.

## Data Availability

Data are contained within the article.
